# Lung epithelial-endothelial-mesenchymal signaling network with hepatocyte growth factor as a hub is involved in bronchopulmonary dysplasia

**DOI:** 10.3389/fcell.2024.1462841

**Published:** 2024-09-03

**Authors:** Yating Sang, Lina Qiao

**Affiliations:** ^1^ Pediatric Intensive Care Unit, West China Second University Hospital, Sichuan University, Chengdu, China; ^2^ Key Laboratory of Birth Defects and Related Diseases of Women and Children, Ministry of Education, Sichuan University, Chengdu, China; ^3^ NHC Key Laboratory of Chronobiology, Sichuan University, Chengdu, China

**Keywords:** hepatocyte growth factor (HGF), bronchopulmonary dysplasia (BPD), growth factors, angiogenesis, epithelial-mesenchymal transition (EMT)

## Abstract

Bronchopulmonary dysplasia (BPD) is fundamentally characterized by the arrest of lung development and abnormal repair mechanisms, which result in impaired development of the alveoli and microvasculature. Hepatocyte growth factor (HGF), secreted by pulmonary mesenchymal and endothelial cells, plays a pivotal role in the promotion of epithelial and endothelial cell proliferation, branching morphogenesis, angiogenesis, and alveolarization. HGF exerts its beneficial effects on pulmonary vascular development and alveolar simplification primarily through two pivotal pathways: the stimulation of neovascularization, thereby enriching the pulmonary microvascular network, and the inhibition of the epithelial-mesenchymal transition (EMT), which is crucial for maintaining the integrity of the alveolar structure. We discuss HGF and its receptor c-Met, interact with various growth factors throughout the process of lung development and BPD, and form a signaling network with HGF as a hub, which plays the pivotal role in orchestrating and integrating epithelial, endothelial and mesenchymal.

## 1 Introduction

Bronchopulmonary dysplasia is one of the most common chronic lung diseases in preterm infants and is a multifactorial condition. Current preventive and therapeutic interventions are insufficient to effectively prevent and treat the arrest of lung development caused by BPD (van de Loo et al., 2024; Ryan et al., 2023; Gilfillan et al., 2021). Each stage of lung development relies on the coordinated interaction between epithelial, endothelial, and mesenchymal cells. The core of late lung development is the formation and maturation of alveoli, a process that necessitates the proliferation, migration, and morphogenesis of distal alveolar epithelial cells, accompanied by the development of the surrounding microvascular network, and also depends on the formation of secondary septa and the support of the extracellular matrix (ECM) (Thébaud and Abman, 2007). It is thought to be regulated by the concerted action of gene expression programs, growth factor signaling, ECM production and maturation (Morrisey and Hogan, 2010; Morrisey et al., 2013; Madurga et al., 2013). In 2001, Steven Abman proposed the “vascular hypothesis” of BPD pathogenesis: during normal lung development, angiogenesis drives alveolar formation, and inhibition of vascular growth itself may directly impair alveolarization (Abman, 2001). Many studies in recent years support this view. For example, Mice lacking platelet endothelial cell adhesion molecule-1 (PECAM1), an endothelial cell (EC) surface molecule that promotes ECs migration and has been implicated in vivo angiogenesis, showed impaired alveolar formation, indicating that ECs play a positive role in alveolar formation (DeLisser et al., 2006). Loss of aerocytes (aCap), alveolar capillary ECs specialized for gas exchange, resulted in hypo-alveolarization (Vila Ellis et al., 2020). Furthermore, EMT is also considered to be one of the significant mechanisms contributing to BPD and subsequent pulmonary fibrosis.

HGF is secreted by cells of mesodermal origin and has powerful mitogenic, motogenic and morphogenic activity on epithelial and endothelial cells (Bardelli et al., 1994). Throughout the developmental processes in humans and animals, HGF is extensively expressed in a variety of tissues and organs, including lungs (Nakamura et al., 2011). In luns, HGF is predominantly expressed by mesenchymal and endothelial cells, while its receptor, c-Met, is primarily located on adjacent epithelial and endothelial cells (Matsumoto et al., 1996). Recent findings have highlighted the close relationship between endogenous HGF and the normal development of pulmonary epithelium, endothelium, and mesenchyme. HGF/c-Met can activate the phosphatidylinositol-3-kinase (PI3K)/Akt signaling pathway and the mitogen-activated protein kinase (MAPK) - ERK1/2 and p27 pathways, promoting various biological effects such as cell proliferation, migration, and angiogenesis (Lee et al., 2008; Takami et al., 2002). HGF is also involved in the regulation of elastin deposition and the inhibition of fibrotic remodeling (Dohi et al., 2000), affecting the formation of the basement membrane in alveolar epithelial cells (Furuyama and Mochitate, 2004). During the repair and regeneration processes following lung injury, HGF plays a crucial role in the pulmonary epithelial, endothelial and mesenchymal (Meng H. F. et al., 2022; Nakamura and Mizuno, 2010). HGF-based gene therapy using mesenchymal stem cells (MSCs) can effectively promote the proliferation of lung epithelial cells and protect them from apoptosis (Wang et al., 2013). HGF can also facilitate the proliferation of pulmonary endothelial cells and the repair of endothelial barrier function following acute lung injury (Hu et al., 2016).

Growth factor signaling disruptions have been linked to the impediment of late-stage lung maturation, constituting one of the mechanisms that can culminate in BPD (Madurga et al., 2013). Such interferences significantly affect the development and maturation of the pulmonary vasculature and alveoli, in addition to the formation of ECM and alveolar septa. In BPD, the expression of multiple growth factors have altered. Notably, a diminished expression has been observed for key factors such as vascular endothelial growth factor (VEGF), angiopoietin (Ang)-1, platelet-derived growth factor (PDGF), and insulin-like growth factor-1 (IGF-1) (Arjaans et al., 2020; Tibboel et al., 2015; Löfqvist et al., 2012; Thébaud et al., 2019). Conversely, HGF, transforming growth factor beta (TGFβ), and basic fibroblast growth factor (bFGF/FGF2) have been detected with elevated expression levels in the bronchoalveolar lavage fluid of preterm infants with BPD and in hyperoxia-induced BPD mice models (Lassus et al., 2003; Aly et al., 2019; Windhorst et al., 2023). Conversely, HGF, transforming growth factor beta (TGFβ), and basic fibroblast growth factor (bFGF/FGF2) have been detected with elevated expression levels in the bronchoalveolar lavage fluid of preterm infants with BPD and in hyperoxia-induced BPD mice models (Lassus et al., 2003; Aly et al., 2019; Windhorst et al., 2023). Currently, HGF is considered a protective factor in BPD, exhibiting cross-talk with multiple growth factors. We summarize the positive role of HGF during lung development for pulmonary epithelium, endothelium, and mesenchyme. We also explore how HGF interacts with other growth factors to enhance alveolar and microvascular density. HGF is proposed to increase alveolar and microvascular density and improve the lung injury structure in BPD through two primary mechanisms: promoting angiogenesis and inhibiting EMT.

## 2 HGF in lung development: promoting lung epithelial, endothelial and mesenchymal development

Lung development is a highly coordinated process that relies on the interactions between pulmonary epithelial, endothelial, and mesenchymal cells. HGF is expressed throughout the entire period of lung development, primarily originating from mesenchymal cells and vascular endothelial cells, while the c-Met receptor is mainly located on pulmonary epithelial cells and vascular endothelial cells. This expression pattern remains fairly constant throughout the developmental process. Lung development in mice begins during the embryonic and pseudoglandular stages, during which the bronchial tree and large parts of the prospective respiratory parenchyma are formed (Calvi et al., 2013). Ohmichi et al. (1998) examined the temporal expression pattern of HGF and c-Met/HGF-R mRNA during lung development, using reverse transcription polymerase chain reaction (RT-PCR). They found that HGF and c-Met mRNA were expressed in the mesenchyme and epithelial tissues, respectively, in the lung buds of embryonic day (E) 13 fetal mice, coinciding with the period when the branching morphogenesis of the pulmonary epithelium is actively occurring (Ohmichi et al., 1998). Kato et al. (2018) confirmed that HGF expression predominates in pulmonary pericytes at postnatal day (P) 7, while Met transcripts are expressed in epithelial cells. Calvi et al. (2013) observed that the HGF ligand is expressed diffusely in the interstitium of the alveolar septum in mice at P14, with c-Met expression localized to alveolar epithelial cells, airway epithelial cells, and a subset of alveolar macrophages. Additionally, Yamamoto et al. (2007) observed a significant reduction in HGF expression in the lungs of mice with pulmonary capillary deficiency, and HGF mRNA was detected in pulmonary endothelial cells at E18.5, indicating that pulmonary vascular endothelial cells are capable of secreting HGF at least during the canalicular phase. Precise balance of angiocrine HGF regulates saccular development in the lung (Yao et al., 2017; Bishop et al., 2023). *In vitro* experiments have also found that alveolar epithelial cells express HGF, but none of the current *in vivo* experiments have found evidence of HGF secretion by alveolar epithelial cells. *In vitro* cultured rat primary alveolar epithelial typeⅡ (ATⅡ) cells, growth arrest-specific protein 6 (Gas6)/Axl or Mer signaling pathways were found to induce RhoA-dependent HGF and c-Met in genes and proteins in ATⅡ cells (Jung et al., 2019).

### 2.1 HGF involves in epithelial branching morphogenesis and alveologenesis

Epithelial branching morphogenesis is a critical phase in early lung development, relying on the coordinated interaction between mesenchyme and epithelium. When mesenchyme is removed from lung rudiments, branching morphogenesis does not occur (Ohmichi et al., 1998). HGF acts as a mesenchymal regulator of lung branching morphogenesis, mediating the signaling between mesenchyme and epithelium. Firstly, mesenchyme-derived HGF, in a dose-dependent manner, can stimulate the proliferation of human bronchial epithelial cells in serum-containing medium through mechanisms involving the MEK-ERK1/2 and PI3K pathways (Takami et al., 2002). More importantly, HGF, in conjunction with other growth factors, promotes the formation of pulmonary epithelial branching morphogenesis. In a mesenchyme-free epithelium culture system, HGF alone did not induce epithelial morphogenesis. However, HGF could synergistically stimulate epithelial branching and morphogenesis induced by acid fibroblast growth factor (aFGF/FGF1) or keratinocyte growth factor (KGF). The explants treated with HGF and KGF, or aFGF increased in size considerably and showed a more complex, uneven budding structure, compared to explants treated with aFGF or KGF alone (Ohmichi et al., 1998). Treatment of E13 mice embryonic lung explants with antisense HGF oligo-DNA or anti-HGF IgG demonstrated a drastic inhibition of epithelial branching and a reduction in end-bud formation, with the stimulatory effects on epithelial branching induced by aFGF almost completely abrogated (Ohmichi et al., 1998).

**FIGURE 1:**
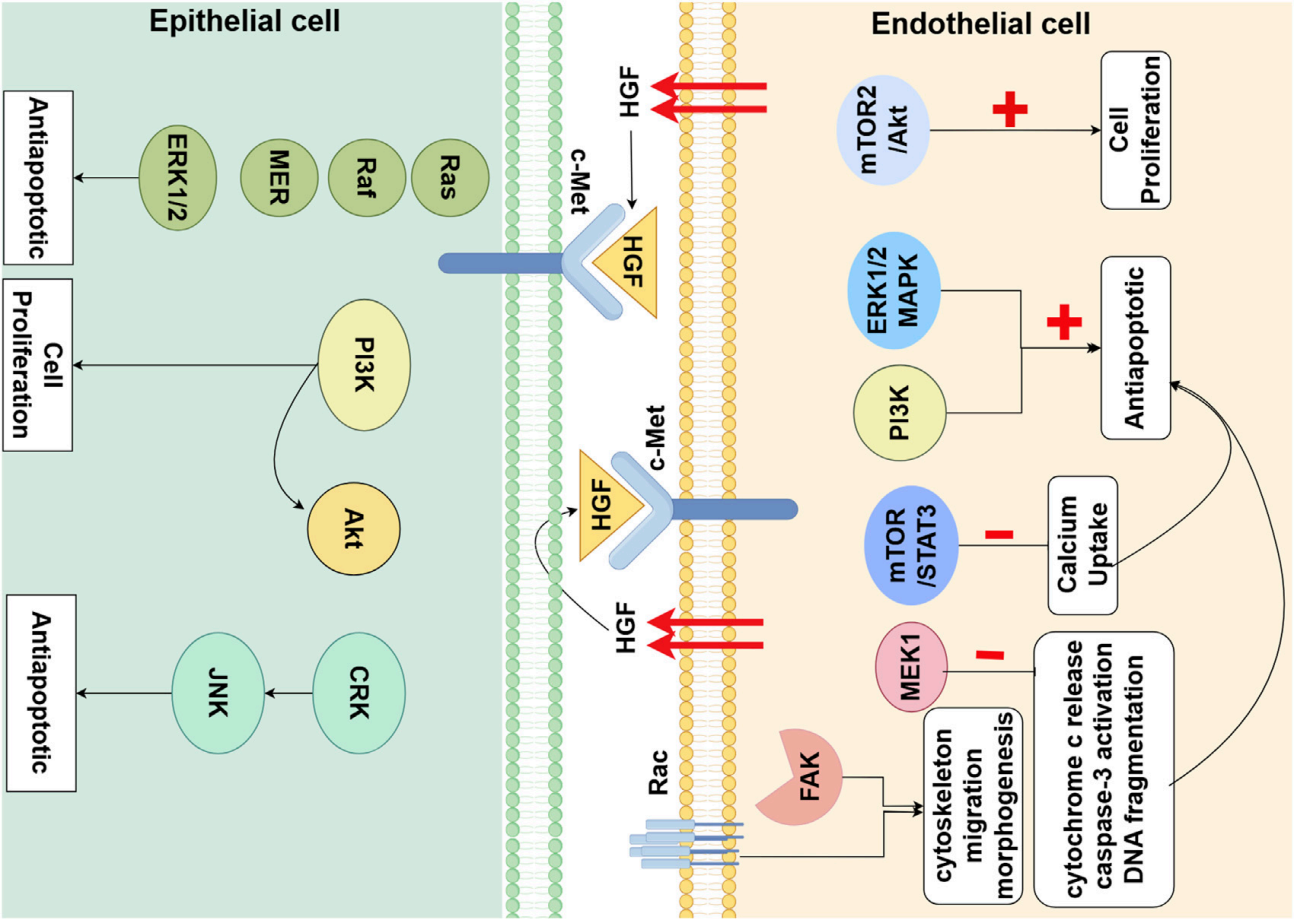
Hepatocyte Growth Factor (HGF) Signaling Pathway in Alveolar Epithelial cells (AECs) and Endothelial Cells (ECs) in Lung Development. HGF, partly secreted by ECs, through paracrine and autocrine mechanisms, binds to the c-Met receptor on AECs and ECs. This interaction triggers a cascade of downstream signaling molecules that primarily promote cell proliferation and anti-apoptotic effects. Additionally, HGF regulates cytoskeletal dynamics, cellular migration, and morphogenesis by activating Rho and FAK (By Figdraw).

During late lung development, mature alveoli form the structural basis for gas exchange. Alveolarization is contingent upon the proliferation and migration of alveolar epithelial cells (Kim et al., 1999). HGF exerts its effects on epithelial cells as a potent mitogen through a paracrine mode of action. *In vitro* experiments have confirmed that HGF induces the proliferation of primary rat ATⅡ cells (Calvi et al., 2013; Shiratori et al., 1995; Seedorf et al., 2016). *In vitro* studies indicate that the HGF-c-Met-ERK1/2 signaling cascade plays an important role in epithelial cell proliferation. Treatment with the neutralizing anti-HGF antibody significantly blocked ATⅡ cells proliferation both *in vivo* and *in vitro* (Chang et al., 2012). The proliferation of ATⅡ cells was also inhibited when isolated primary ATⅡ cells were co-cultured with c-Met inhibitor SU11274 (Zeng et al., 2016). The deficient expression of Met in P14 mice ATⅡ cells results in impaired airspace morphology and is associated with reduced abundance and survival of ATⅡ cells. Conversely, the enhancement of HGF signaling in pulmonary epithelial cells can induce the activation of ERK, JNK, and Akt, reduce apoptosis of alveolar epithelial cells, and ameliorate pathological airspace enlargement (Calvi et al., 2013) (Figure 1).

### 2.2 HGF promotes the formation and maturation of alveolr capillary network

Alveolarization is contingent upon the formation and maturation of the microvascular network surrounding the alveoli (Thébaud and Abman, 2007). The development of this microvascular network is fundamental to the process of alveolar formation. HGF works in concert with other pro-angiogenic factors to collectively stimulate the proliferation and migration of pulmonary vascular endothelial cells, inducing angiogenesis and vascular maturation. It ultimately results in the formation of a single-layer capillary network around the alveoli for efficient gas exchange. Firstly, co-cultures of mesenchymal cells and pulmonary microvascular endothelial cells (PMVECs) have revealed that HGF induces PMVEC proliferation in a manner dependent on the mTORC2/Akt signaling pathway (Meng et al., 2021). Binding of the Met receptor by HGF triggers the activation of two major antiapoptotic pathways: the ERK1/2 MAPK pathway and the PI3K pathway. HGF activation of MEK1 alone is sufficient to abrogate Ang2-induced endothelial cells apoptosis, by inhibiting cytochrome c release, caspase-3 activation, and DNA fragmentation (Lee et al., 2010). Secondly, it can directly promote angiogenesis by enhancing intracellular signaling molecules involved in cytoskeletal remodeling, cell migration, and morphogenesis, such as focal adhesion kinase (FAK) and Rac (Sulpice et al., 2009). HGF directly stimulates fetal pulmonary artery endothelial cell growth and tube formation, which is attenuated by treatment with JNJ-38877605, a c-Met inhibitor (Seedorf et al., 2016) (Figure 1).

Most importantly, HGF synergizes with other pro-angiogenic growth factors to collectively promote the formation and maturation of new blood vessels. VEGF is the most critical pro-angiogenic factor, regulated by the upstream hypoxia-inducible factor (HIF) −1, stimulating the migration and proliferation of vascular endothelial cells to sprout new blood vessels from pre-existing ones (Laddha and Kulkarni, 2019). However, VEGF alone promotes the formation of immature, highly permeable vessels. HGF has a synergistic effect with VEGF in promoting the formation and maturation of new blood vessels. The combined use of both can balance the permeability of endothelial cells in new blood vessels by regulating the RhoA/Rac1 pathway, reducing capillary leakage and forming more stable, less regressive mature blood vessels. RhoA and Rac1 are key regulators controlling endothelial barrier function. Rho enhances endothelial cell permeability, while Rac1 opposes it, balancing the permeability of the vascular barrier (Nagy and Senger, 2006). *In vitro* experiments have found that, HGF selectively activates Rac1, induces the extension of lamellipodia, and promotes a branching phenotype of capillary-like networks (Meng S. et al., 2022; Yang et al., 2015; Meng et al., 2019; Birukova et al., 2008). VEGF selectively upregulates Rho activity, stimulates the formation of stress fibers, leading to the formation of tubular networks and enhancing endothelial cell permeability (Nagy and Senger, 2006). VEGF and HGF regulate distinct morphogenic aspects of the cytoskeletal remodelling that are associated with the preferential activation of Rho or Rac respectively, and induce structurally distinct vascular-like patterns *in vitro* in a Rho- or Rac-dependent manner (Sulpice et al., 2009). Ang1 is also an important pro-angiogenic factor that mediates endothelial cell survival through the activation of the PI3K/AKT and MAPK pathways, and promotes angiogenesis and vascular maturation, relying on nitric oxide (NO) derived from endothelial cells (Stenmark and Abman, 2005; Liu et al., 2008). In lung vascular development, HGF and Ang1 also exhibit synergistic effects. HGF and Ang1 can jointly inhibit VEGF-mediated endothelial cell calcium uptake, thereby preventing VEGF-mediated endothelial hyperpermeability, but through different mechanisms. HGF inhibits the calcium (Ca) uptake of endothelial cells through the mTOR/STAT3 signaling pathway (Meng S. et al., 2022). Ang1 blocks VEGF-induced TRPC1-dependent Ca2+ influx through the interaction between IP3R and TRPC1 (Jho et al., 2005). Another significant potent pro-angiogenic factor is bFGF secreted by mesenchymal cells. Kaga et al. found that HGF and bFGF can jointly regulate the balance of endothelial cell and vascular smooth muscle cell (VSMC) proliferation and migration, which would otherwise lead to immature vascular leakage (Kaga et al., 2012). HGF primarily increases the number of endothelial cells and promotes their migration, while bFGF significantly increases the number of VSMCs. Moreover, HGF does not activate the essential transcription factor for inflammation, NF-κB, nor does it recruit inflammatory cells, thereby inducing the formation of low-permeability new blood vessels. In contrast, bFGF significantly activated NF-κB and upregulates gene expression of its downstream inflammation-related cytokines, such as interleukin-8 (IL-8) and monocyte chemoattractant protein-1 (MCP-1), in VSMCs, leading to increased vascular permeability (Kaga et al., 2012).

### 2.3 HGF recruits alveolar epithelial cells and endothelial cells to build the basement membrane, participating in the formation of secondary septa in the lung mesenchyme

The formation of alveolar septa compartmentalizes the saccular/alveolar spaces into smaller units for gas exchange, a process that depends on the close interplay between alveolar epithelial cells, the pulmonary microcapillary network, and mesenchymal myofibroblasts (Whisler et al., 2023). In this process, initially, pulmonary microvascular endothelial cells (PMECs) activate integrin β1 in a Rap1 (a small GTPase)-dependent manner, inducing the recruitment of Collagen type IV (Col4) into the ECM, which promotes cell adhesions into the ECM and the formation of cadherin-mediated cell-cell junctions (Watanabe-Takano et al., 2024). Subsequently, these basement membranes serve as a scaffold for myofibroblasts (MYFs). MYFs and pericytes upregulate the expression of microelastic-related genes, such as Col1a1, Col3a1, and Col1a2 (Mammoto and Mammoto, 2019). This upregulation recruiting elastin and collagen deposition at the tips of the alveolar septa, leading to an increase in the stiffness of the peri-alveolar interstitium, and inducing changes in mechanical forces and the promotion of secondary septum formation (Zhou et al., 2018; D’Urso and Kurniawan, 2020). During alveologenesis, myofibroblasts contract to form secondary septa and modulate the mechanics of vascularization. This process relies on the activation of mechanical signaling pathways, including the activation of myosin light chain kinase (MLCK) and the nuclear localization of Yes-associated protein (YAP) (He et al., 2021; Li R. et al., 2020).

**FIGURE 2:**
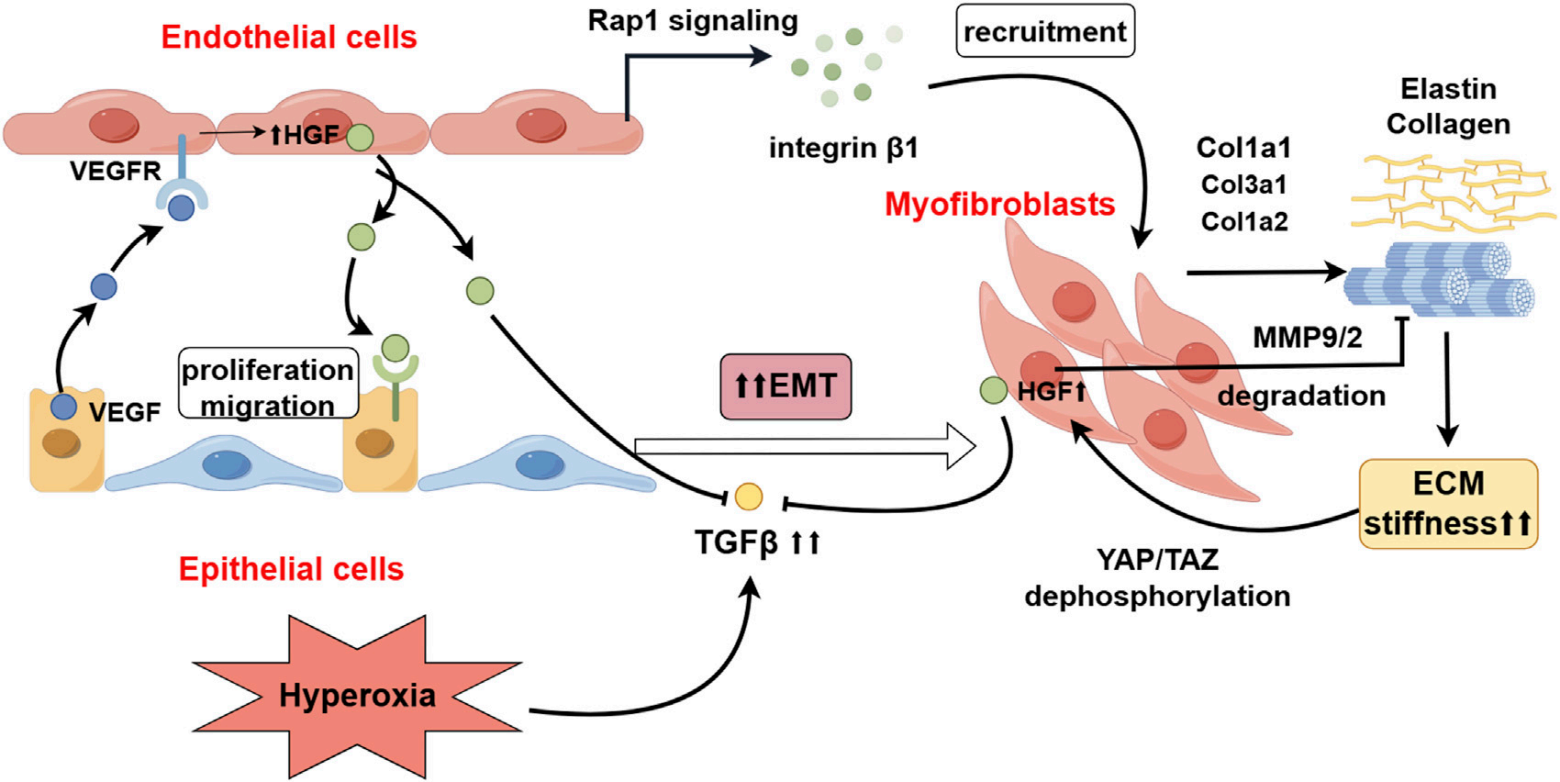
The Role of HGF in the Crosstalk between Lung Mesenchyme-Endothelium-Epithelium. HGF, derived from endothelial cells, activates the Rap1 signaling pathway, recruiting myofibroblasts (MYF) and promoting the deposition of extracellular matrix (ECM) proteins, including elastin and collagen. Increased ECM stiffness, through mechanosignaling, leads to the dephosphorylation and activation of YAP/TAZ, thereby stimulating the secretion of HGF by myofibroblasts (MYFs). Hyperoxia upregulates the expression of TGFβ, which promotes epithelial-mesenchymal transition (EMT) in epithelial cells. HGF inhibits the occurrence of EMT by antagonizing the expression of TGFβ and degrading ECM-related proteins (By Figdraw).

RNA-seq analysis has demenstrated that lung fibroblasts produce high levels of HGF (Kosyakova et al., 2020). As previously described, during the process of alveolarization, HGF recruits endothelial cells to form the alveolar capillary network surrounding the alveoli. Similarly, HGF also recruits alveolar epithelial cells to aggregate at the basement membrane during alveolarization. Iin vitro experiments demonstrated that HGF stimulated the proliferation and migration of alveolar epithelial cells on the basement membrane substratum in a dose-dependent manner (Kobayashi et al., 2006). Additionally, HGF mediates the recruitment and migration of myofibroblasts towards endothelial cells induced by Ang1 (Furuyama et al., 1999). However, it has been found that HGF promotes the degradation of ECM components, such as laminin, collagen, and fibronectin, by inducing both the proteolytic activities and protein levels of matrix metalloproteinases (MMP) −9 and urokinase plasminogen activator (uPA), thus inhibiting the formation of basement membrane (Furuyama and Mochitate, 2004). In contrast, TGFβ1 significantly enhanced the synthesis of these basement membrane constituents in a dose-dependent manner (Furuyama et al., 1999). HGF and TGFβ mutually negatively regulate each other, balancing the synthesis and degradation of basement membrane components to prevent excessive ECM deposition. ECM modulates the expression of HGF by pericytes through mechanical signal transduction. High ECM stiffness transmits mechanical signals to RhoA GTPase by increasing cytoskeletal tension, which in turn inactivates LATS1/2 kinase in the Hippo pathway, leading to the dephosphorylation and nuclear localization of YAP/TAZ, thereby regulating the expression of HGF by pericytes (Cai et al., 2021; Dasgupta and McCollum, 2019). In the lungs of P7 mice with double-knockout mutations of Yap1/TAZ, the expression of HGF in pericytes was significantly reduced (Kato et al., 2018). Specifically induced c-met gene inactivation (c-met^SP-C-Δ/Δ^) in epithelial cells starting from E14.5, the c-met^SP-C-Δ/Δ^ mice lungs displayed impaired saccular development and exhibited enlarged distal airspaces with few primary septae (Yamamoto et al., 2007). These data demonstrate that HGF signaling between lung epithelial-endothelial-mesenchymal cells is indeed necessary for normal septae formation and alveolarization (Figure 2).

## 3 HGF in BPD: ameliorating the lung injury structure of BPD by two mechanisms

In humans, from the 23rd week of gestation through the late canalicular and terminal saccular stages up to the end of the fetal period, and in mice from E17.5 to P5, lung development is at a critical stage for the formation and maturation of alveoli and the capillary network (Calvi et al., 2013). Due to premature exposure to the extrauterine environment, preterm infants experience an arrest in the lung developmental program, leading to persistent structural lung damage (Benjamin et al., 2021), characterized by a reduction in pulmonary vascular density and surface area, a decrease in the number and diameter of alveoli, and abnormal thickening of the alveolar septa (Thébaud et al., 2019; Mourani and Abman, 2013). Decreased levels of HGF are detected in tracheal aspirate fluid (TAF) samples from infants with BPD. The lower the concentration of HGF, the more severe the degree of BPD exhibited by preterm infants (Lassus et al., 2003). Padela et al. (2005) observed a significant upregulation of HGF and c-Met expression levels in lungs of mice (P14) with BPD induced by hyperoxia. These findings suggest that HGF may be one of the significant influencing factors in BPD. These findings suggest that HGF may be one of the significant influencing factors in BPD. Studies have confirmed that HGF ameliorates the severity of BPD by protecting alveolar epithelium and microvessels from hyperoxic injury, promoting the formation of alveolar septa, and improving alveolar simplification. Calvi et al. (2013); Yamamoto et al. (2007) selectively induced specific inactivation of the c-Met gene in mice alveolar epithelial cells, with results showing a reduction in ATⅡ cell abundance and alveolar number, thickening of alveolar septa, and abnormal alveolar expansion, exhibiting pathological changes similar to BPD-injured lungs. Ohki et al. (2009) reported a marked improvement in alveolar simplification and an increase in the number of microvessels around the alveoli after administering recombinant human HGF (rhHGF) to BPD mice induced by hyperoxia. Injection of HGF neutralizing antibody or truncated soluble c-Met receptor in newborn mice also resulted in alveolar simplification structures similar to those seen in BPD (Padela et al., 2005). Transplantation of human umbilical cord blood-derived mesenchymal stem cells (HUC-MSCs) into newborn rats continuously exposed to 90% oxygen reduced cell apoptosis and inflammation, and increased the secretion of HGF and VEGF, which improved the hyperoxic damage to alveolar formation and angiogenesis. The protective effect of HUC-MSCs against hyperoxic lung injury was positively correlated with the levels of HGF and VEGF they produced (Ahn et al., 2015). Additionally, as previously described, the HGF/c-Met signaling pathway can reduce the susceptibility of cells to hyperoxic injury during alveolar formation by activating high expression of STAT3 (Calvi et al., 2013). Therefore, HGF can be identified as a protective factor against lung injury in BPD. However, whether the expression of HGF is upregulated or downregulated in the hyperoxia-induced BPD mice model may depend on the induced oxygen concentration and the duration of continuous hyperoxic exposure. Most experiments have found that the expression of HGF is upregulated in the lungs of BPD mice models. An exception to this is the study by Ahn et al. (2015), who exposed newborn rats to 90% oxygen and found a significant reduction in pulmonary HGF levels on postnatal days 7 and 14. It is speculated that this may be due to the continuous high oxygen concentration causing severe endothelial injury, thereby reducing the production of HGF from vascular endothelial cells.

### 3.1 HGF interacts with other growth factors to promote neovascularization and drive alveolar formation

Recent studies on BPD also support the idea of the “vascular hypothesis”, which suggests that impaired vascular development may be a key factor in BPD-associated alveolar simplification. By comparing the lung structure of BPD mice and wild-type mice on different days in the early postnatal period, it was found that the reduction in pulmonary capillary endothelial surface area in BPD lungs precedes the reduction in alveolar epithelial surface area, suggesting that impaired vascular development precedes impaired alveolar formation (Appuhn et al., 2021).

HGF has been identified as mediators of reciprocal communication between the epithelium and endothelium. As previously mentioned, vascular endothelial cells are one of the important sources of HGF. These cells can stimulate their own proliferation and angiogenesis through autocrine secretion of HGF and also promote cell proliferation and alveolar formation in ATⅡ cells through paracrine actions (Seedorf et al., 2016). HGF, dependent on the mTORC2/Akt signaling pathway, can directly induce the proliferation of PMVECs (Meng et al., 2021) and activate the ERK1/2 MAPK and PI3K signaling pathways to inhibit endothelial cell apoptosis (Lee et al., 2010). After treating LPS-induced damaged endothelial cells with HGF, it was found that HGF protected the endothelium via the suppression of reactive oxygen species (ROS) production and intracellular calcium uptake via the mTOR/STAT3 pathway, thereby alleviating endothelial oxidative stress injury and cell apoptosis (Meng S. et al., 2022). HGF could promote the expression of endothelial junction proteins such as VE-cadherin and occludin, and decrease endothelial paracellular and transcellular permeability during LPS-induced endothelial dysfunction, with the involvement of the mTORC2/Akt and mTORC1 (raptor) pathways, to maintain the integrity of the pulmonary vascular endothelial barrier (Arjaans et al., 2020; Meng et al., 2021).

It has been shown that decreased proangiogenic and increased antiangiogenic factors are associated with high risk for the development of BPD (Arjaans et al., 2020; Mandell and Abman, 2017). HGF is a typical proangiogenic factor. VEGF is recognized as the most important growth factor that can improve BPD. Recent studies have found that the role of VEGF in improving the structure of lung injury is partially dependent on the downstream HGF/c-Met signaling pathway. Yamamoto et al. found that in mice with selective inactivation of the VEGF-A gene in respiratory epithelium, HGF expression on pulmonary endothelial cells was reduced, accompanied by an almost complete absence of pulmonary capillaries and defects in the formation of primary septa (Yamamoto et al., 2007). Seedorf et al. injected the VEGF receptor inhibitor SU-5416 into newborn rats and observed a reduction in distal lung vascular density and alveoli simplification structure, exhibiting characteristics of BPD, but treatment with rhHGF was able to directly stimulate angiogenesis and increase the number of alveoli (Seedorf et al., 2016). Previous studies have demonstrated that endothelial nitric oxide synthase (eNOS)/NO is one of the downstream signaling molecules of VEGF, mediating VEGF-induced endothelial cell proliferation and angiogenesis (Laddha and Kulkarni, 2019; Han et al., 2004). However, in the absence of eNOS/NO, HGF may serve as a significant compensatory mechanism for the VEGF signaling pathway. HGF phosphorylates eNOS not only through the PI3K/Akt pathway in a Ca(2+)-sensitive manner in vascular endothelial cells (Makondo et al., 2003), but also partially through the MAPK pathway (Uruno et al., 2004). Compared with VEGF, HGF is more potent in both NO production and eNOS phosphorylation. When eNOS gene knockout (eNOS^−/−^) mice are placed in a hyperoxic environment, it is observed that the levels of HGF and c-Met proteins in the lungs are significantly decreased compared to eNOS^+/+^ mice. Treatment with exogenous recombinant human VEGF (rhVEGF) can upregulate the expression levels of HGF and c-Met proteins in eNOS^−/−^ mice (Seedorf et al., 2016), indicating that the HGF/c-Met signaling transduction may be impaired after the knockout of the eNOS gene. These studies suggest that VEGF exerts pro-angiogenic effects through downstream eNOS/NO signaling molecules or the HGF/c-Met non-eNOS-dependent signaling pathway. These two pathways intersect and influence each other. As previously mentioned, it has been confirmed during lung development that HGF synergistically promotes angiogenesis and maturation of new blood vessels with Ang1 and bFGF, but there is currently a lack of research on the relationship between HGF and Ang1/bFGF in BPD models. TGFβ is also an important intra-pulmonary regulatory growth factor for angiogenesis, and its role in promoting or antagonizing angiogenesis depends on different downstream pathways. Hyperoxia activates the TGFβ-ALK5-Smad2/3 signaling transduction in pulmonary endothelial cells, to inhibit lung angiogenesis (Lee et al., 2020). HGF can antagonize the production of TGFβ (mechanism see below).

### 3.2 HGF inhibits EMT by suppressing TGFβ1 expression and degrading ECM-related proteins

Epithelial-mesenchymal transition refers to the process by which epithelial cells lose their polarity and cell adhesion characteristics, acquiring the phenotype and behavior of mesenchymal cells (Yang et al., 2020). The dissolution of epithelial cell-cell junctions or pathological remodeling of ECM are potential triggers for EMT (Lamouille et al., 2014). In hyperoxia-induced BPD rats, evidence of excessive EMT in ATⅡ cells has also been observed (Yang et al., 2017). In the ATⅡ cells of BPD rats, the co-expression of surfactant protein C (SPC) and alpha-smooth muscle actin (a-SMA) can be observed, along with characteristic ultrastructural changes of EMT, including vacuolization of some ATⅡ cells and a significant increase in actin microfilaments (Yang et al., 2014). Once ATⅡ undergo EMT and transdifferentiate into fibroblasts, they lose the ability to form alveoli and repair lung injury (Yang et al., 2016). Concurrently, in BPD rats, it has been found that the levels of E-cadherin protein, which is related to cell adhesion, are significantly reduced, while the expression of ECM-related proteins, including a-SMA protein and N-cadherin, is abnormally elevated (Yang et al., 2014; Li C. et al., 2020), leading to changes in the mechanical stress of the local alveolar microenvironment and thus affecting the formation of alveoli and secondary septa.

HGF is pivotal to inhibit the occurrence of EMT, suppressing the onset of EMT after lung injury through two mechanisms: (1) HGF directly inhibits TGFβ1-mediated EMT. Overexpression of TGFβ1 has been found in hyperoxia-induced BPD mice (Aly et al., 2019), and TGF-β1 protein is primarily expressed in the walls of the distal air spaces and in bronchiolar epithelium and vascular endothelial cells. From P3 to P14, the pattern of expression of TGF-β1 protein at each time point was consistent with that of elastin. TGF-β1 is considered an important growth factor that induces EMT in injured lungs. Hyperoxia can upregulate the expression of TGFβ1, to downregulate the expression of the E-cadherin gene and increase the production of hyaluronic acid (HA), leading to abnormal deposition of fibulins and elastin, hindering normal matrix remodeling, and resulting in increased local ECM stiffness surrounding the alveoli (Han et al., 2015). Pathological increases in ECM stiffness can lead to alterations in local mechanical stress within the alveoli, activating the nuclear translocation of YAP/TAZ in myofibroblasts through both Hippo-dependent and -independent pathways. This activation can induce the expression of downstream TEAD transcription factors and target genes such as SERPINE1 (which encodes PAI-1), promoting EMT and pulmonary fibrosis (Heise et al., 2011; Park and Kwon, 2018; Zhao et al., 2008). However, it also stimulates the expression of HGF in pericytes. HGF, dependent on the MAPK signaling pathway, can stimulate the expression and nuclear translocation of the TGFβ negative regulatory factors Smad7 and the Smad ubiquitin regulatory factor 1 (Smurf1), blocking the TGFβ1-Smad2/3 signaling transduction, thereby inhibiting the expression of type Ⅰ collagen and fibronectin induced by TGF-β1, and suppressing the occurrence of EMT (Chakraborty et al., 2013). (2) HGF induces the degradation of abnormally deposited ECM proteins and mediates the apoptosis of myofibroblasts. HGF can upregulate the expression of MMP-9/-2, degrading the ECM proteins surrounding myofibroblasts (Mizuno et al., 2005), thereby downregulating the mechanical transmission signals caused by increased ECM stiffness, and subsequently inhibiting the formation and transcriptional activity of the YAP/TAZ-TEAD complex (Moure et al., 2024) (Figure 2). When myofibroblasts lose the support of the ECM, they activate the intrinsic death program, known as anoikis (Chakraborty et al., 2013).

## 4 Conclusion

HGF plays an essential role in the normal development of the alveolar and microvascular networks in lungs. HGF is involved in pulmonary epithelial branching morphogenesis and the promotion of the formation of the microvascular network surrounding the alveoli, linking the pulmonary epithelial-endothelial-mesenchymal components during distal lung development and promoting the formation of alveolar septa and alveolar units. In BPD, HGF interacts with other growth factors to improve the alveoli simplified structure by promoting angiogenesis and inhibiting the occurrence of EMT, thereby alleviating or reversing the severity of lung injury in BPD. HGF has synergistic or antagonistic effects with various growth factors in lung development, but in BPD research, the focus is mainly on the relationship between HGF and VEGF. In fact, in other disease models, relationships between other growth factors and HGF have also been identified, such as Ang1, FGF, PDGF, etc. The expression of these growth factors also altered significantly in BPD. The expression of Ang1 is downregulated in BPD (Tibboel et al., 2015). In mice after pulmonary resection, studies have found that blood vessels induced by HGF alone will regress due to a decrease in angiogenic stimulation (Sakamaki et al., 2002). The combined treatment of rats with pulmonary arterial hypertension with HGF and Ang1 can better improve the effective perfusion of newly formed microvessels and the integrity of endothelial cell adhesion junctions, exhibiting a more mature and stable phenotype of newly formed blood vessels (Miao et al., 2021). In a bleomycin-induced pulmonary fibrosis mouse model, Ang-(1-7) was found to directly inhibit TGFβ1-induced EMT in alveolar epithelial cells by disrupting the TGFβ1-Smad signaling pathway (Shao et al., 2019). In the bronchoalveolar lavage fluid of children with BPD, elevated expression of bFGF and reduced expression of PDGF were observed (Arjaans et al., 2020; Windhorst et al., 2023; Popova et al., 2014). In a mouse model of limb ischemia, bFGF was found to directly stimulate the expression of HGF mRNA through the p42/44 MAPK pathway in the early stage, and to indirectly induce HGF expression by stimulating the secretion of endogenous PDGF-AA in the later stage (Onimaru et al., 2002). In this model, PDGF can induce the production of HGF in pulmonary mesenchymal cells through downstream Ras and p70S6K signaling molecules. PDGF is an important growth factor produced by pulmonary mesenchymal cells and can promote the production of elastin-related proteins in pulmonary mesenchyme through the PI3K/AKT pathway (Caldeira et al., 2021), and promote the formation of alveolar septa (Liu et al., 2023). Mice with induced deficiency of PDGF expression in myofibroblasts exhibit reduced and abnormally distributed elastin mRNA, as well as decreased levels of TGFβ mRNA (Li et al., 2018). PDGF may have synergistic effects with HGF in BPD to inhibit TGFβ and EMT. In summary, HGF interacts with multiple growth factors, constructing a signaling network between epithelial, endothelial, and mesenchymal cells, and collectively regulates the development of alveoli and pulmonary vasculature, participating in lung injury repair. This will provide new insights for future research on the pathogenesis and treatment of BPD.
